# Complete mitochondrial genome of the sea-pen, *Cavernularia obesa* (Valenciennes, 1850) (Octocorallia: Veretillidae)

**DOI:** 10.1080/23802359.2022.2163596

**Published:** 2023-01-08

**Authors:** Hye-Jin Eom, Jae-Sung Rhee

**Affiliations:** a Department of Marine Science, College of Natural Sciences, Incheon National University, Incheon, South Korea; b Research Institute of Basic Sciences, Incheon National University, Incheon, South Korea; c Yellow Sea Research Institute, Incheon, South Korea

**Keywords:** Sea-pen, mitogenome, veretillidae, cavernularia obesa

## Abstract

Here, we sequenced and annotated the complete mitochondrial genome for the sea-pen, *Cavernularia obesa* (Valenciennes, 1850). The complete mitogenome of *C. obesa* is 18,641 bp, with 34.7% of GC ratio. The mitogenome comprises 13 protein-coding genes (PCGs), two ribosomal RNA (rRNA) genes, 22 transfer RNA (tRNA) genes, and a non-coding region. Phylogenomic analysis based on 19 in-group taxa belonging to the orders Alcyonacea and Pennatulacea has congruent with published phylogenetic relationship for Octocorallia, which *C. obesa* was grouped to members of the Pennatulacea. This mitogenome resource will be useful for future phylogenetic studies of water fleas.

Octocorallia (Cnidaria: Anthozoa) includes three orders, Alcyonacea, Helioporacea, and Pennatulacea that are comprising over 3,500 species of blue corals, soft corals, gorgonians, and sea pens (Bayer 1981; Williams and Cairns 2013). The benthic Cnidarians, octocorals are widely distributed from shallow environment to deep-sea across all climate zones, providing shelter for a particular invertebrate fauna and fish communities (McFadden et al. 2010). In general, they are soft-bodied habitat-forming organisms with unique characteristics of eightfold radial symmetry such as eight tentacles and eight internal mesenteries (Watling et al. 2011). Members of the order Pennatulacea are commonly known as colonial marine cnidarians, sea pens. Sea pens are widely distributed in soft sediments from polar seas to the equatorial tropics by using root-like peduncles to anchor themselves in sandy or muddy substrate (Williams 2011). Although the information on their geographic distribution, habitats in mesophotic environments, and diversity has been consistently studied, knowledge about the molecular phylogenetic relationship is nascent due to the absence of whole mitogenome resources.

An individual organism of *C. obesa* was isolated from Muui Island (37°03′N, 126°04′W; Incheon, South Korea). Morphometric characteristics including the internal structure, distribution in the coastal regions of South Korea, and habitat information of our sample were carefully compared with the information described in the database of the National Institute of Biological Resources of South Korea (National Institute of Biological Resources 2022) and previous studies (Song and Lee 1998; Veena and Kaladharan 2013). The specimen was deposited in the fish collection at the Research Institute of Basic Sciences of Incheon National University (Specimen ID: Cnidaria-03; https://www.inu.ac.kr/user/indexMain.do?siteId=ribs; Dr. Sang-Eun Nam; se_nam2@inu.ac.kr). Total genomic DNA was isolated from inner layer tissue of the single specimen using a DNeasy Blood and Tissue kit (Qiagen, Hilden, Germany). Three mitochondrial genes, *COI*, *cob*, and *rrnL* were targeted as initial amplification for a long-PCR procedure. The genes were amplified by PCR with the conservative primer sets (Folmer et al. 1994). Long fragments were amplified to obtain sequences in the gaps between partial genes with the followed long-PCR condition: 40 cycles of 98 °C for 25 s and 68 °C for 12 min in a 50 μL reaction mixture containing 30.5 μL distilled water, 5 μL 10 × LA PCR buffer II (TaKaRa, Japan), 8 μL dNTP (4 mM), 5 μL of each primer (5 μM), 0.5 μL LA Taq polymerase (2.5 U), and 1 μL of *C. obesa* genomic DNA. The resulting circular sequence was confirmed using MITOS2 (Bernt et al. 2013) and tRNAscan-SE 2.0 (Lowe and Eddy 1997) and multiple alignments with sea pens’ mitogenomes confirmed the identity of these genes.

The complete circular mitogenome of *C. obesa* is 18,641 bp in length (GenBank Accession no. OK149222), containing of 13 PCGs, 22 tRNAs, two rRNAs, and one non-coding conserved region presumed to be the control region. The nucleotide composition was highly biased toward A + T nucleotides (65.3%), with percentages of A, T, C, and G were 30.6%, 34.7%, 15.9%, and 18.8%, respectively. The overall genome architecture of the *C. obesa* mitogenome is conserved and similar to other mitogenome sequences of the Octocorallia. We constructed the phylogenetic topology of 19 members belonging to the order Alcyonacea and Pennatulacea using the concatenated nucleotide sequences of 13 PCG sequences, with two species as an outgroup (Figure 1). JModelTest ver. 2.1.10 (Darriba et al. 2012) was used to select the best substitution model and the GTR + G + I model was applied to perform a maximum-likelihood analysis using PhyML 2.4.5 (Guindon and Gascuel 2003) with 1000 bootstrap replicates. Of the members of Pennatulacea, *C. obesa* is closely related to *Virgularia mirabilis* (Virgulariidae). Overall topology of the members of Alcyonacea and Pennatulacea was monophyletic with high bootstrap values, although each family was also monophyletic. Previously, the families Veretillidae has been highlighted to be the one of earliest-diverging families in sea-pen phylogeny (Kükenthal and Broch 1911; Niedermeyer 1913; Williams 1995), as shown in our analysis. However, a recent phylogenetic study conducted with *mtMutS* and *ND2* sequences suggested that the family Veretillidae is to be derived taxa and not in an ancestral position (Kushida and Reimer 2019). Thus, further molecular resources of taxonomic markers are needed for the understanding of the origin and evolutionary history of sea pens.

**Figure 1.**
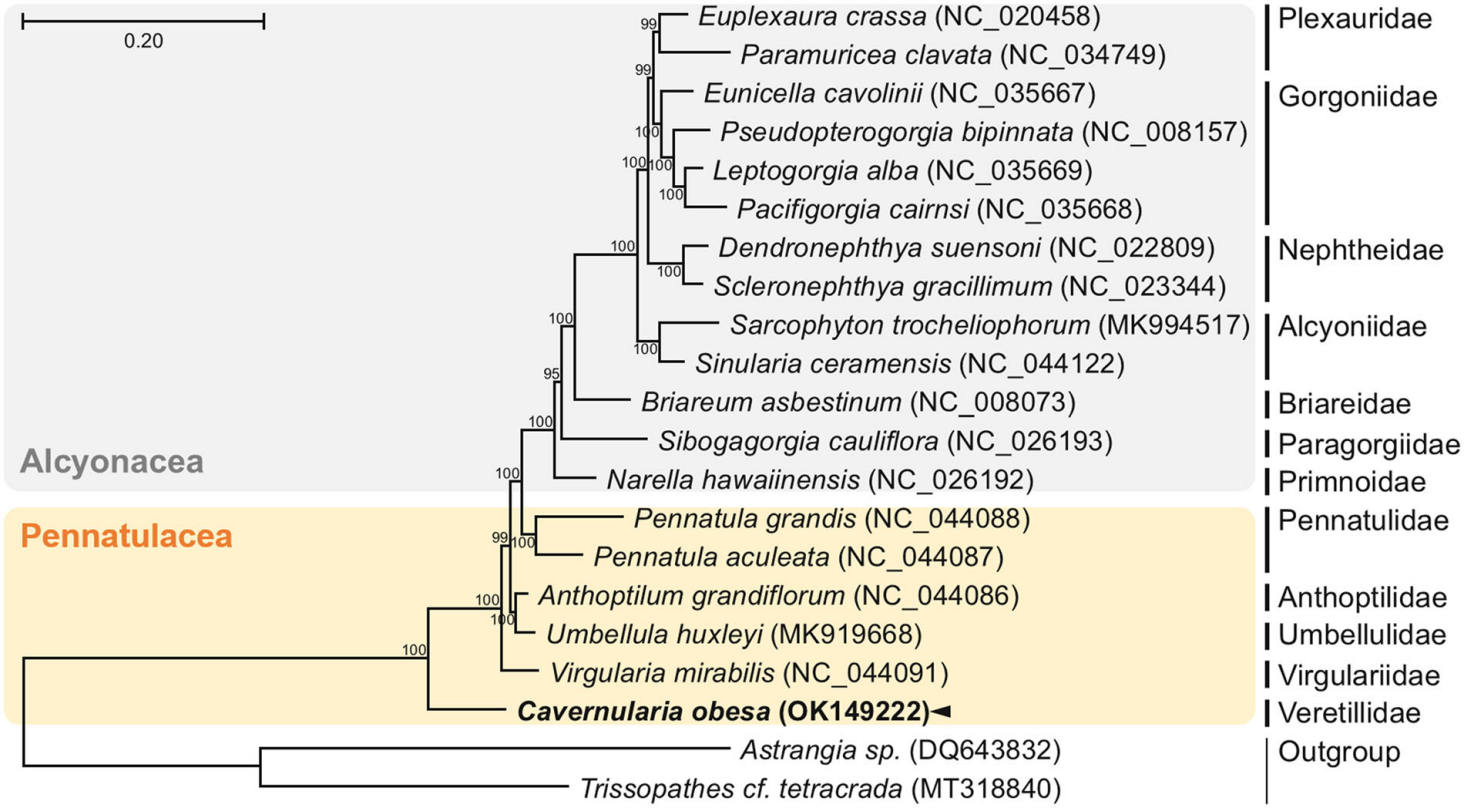
Maximum-likelihood (ML) phylogeny of five published mitogenomes of the order Pennatulacea including *C. obesa* and 13 registered mitogenomes of the order Alcyonacea based on the concatenated nucleotide sequences of protein-coding genes (PCGs). Numbers on the branches indicate ML bootstrap percentages. DDBJ/EMBL/Genbank accession numbers for published sequences are incorporated. The black arrow means the sea-pen analyzed in this study.

## Ethical approval

This study did not involve endangered or protected species, and the sea pen was collected under the guide line of National Institute of Ecology. Field studies have been carried out in accordance with guidelines and comply with local legislation. One individual was collected from the Muui Island (37°03′N, 126°04′W; Incheon, South Korea).

## Data Availability

As we sequenced the complete mitogenome using conventional PCR method and no NGS platform was conducted, BioProject, SRA and Bio-Sample accession numbers are not required. The data that support the findings of this study are openly available in the National Center for Biotechnology Information (NCBI) at https://www.ncbi.nlm.nih.gov, accession number OK149222.
